# {2-Morpholino-*N*-[1-(2-pyrid­yl)ethyl­idene]ethanamine-κ^3^ *N*,*N*′,*N*′′}bis­(thio­cyanato-κ*N*)zinc(II)

**DOI:** 10.1107/S1600536810053778

**Published:** 2011-01-08

**Authors:** Nura Suleiman Gwaram, Nurul Azimah Ikmal Hisham, Hamid Khaledi, Hapipah Mohd Ali

**Affiliations:** a Department of Chemistry, University of Malaya, 50603 Kuala Lumpur, Malaysia

## Abstract

The asymmetric unit of the title compound, [Zn(NCS)_2_(C_13_H_19_N_3_O)], contains two crystallographically independent mol­ecules. In each mol­ecule, the Zn^II^ ion is five-coordinated by the *N,N′,N"*-tridentate Schiff base and the N atoms of two thio­cyanate ligands in a distorted square-pyramidal geometry. The two mol­ecules differ mainly in the deviations from the ideal geometry, with τ values of 0.14 and 0.33. In the crystal, inter­molecular C—H⋯S hydrogen bonds are observed. An intra­molecular C—H⋯N hydrogen bond occurs in one of the independent mol­ecules.

## Related literature

For the crystal structures of similar zinc complexes, see: Cai (2009 ▶); Chen *et al.* (2005 ▶). For a description of the geometry of complexes with five-coordinate metal atoms, see: Addison *et al.* (1984 ▶).
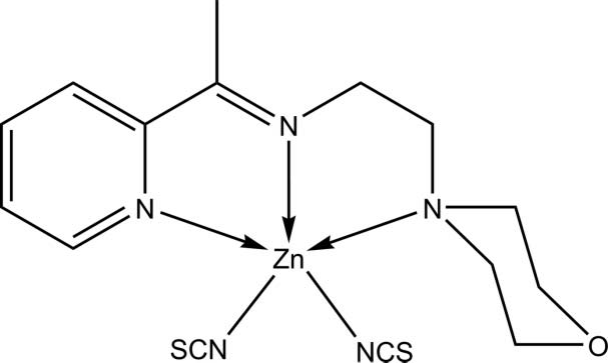


## Experimental

### 

#### Crystal data

[Zn(NCS)_2_(C_13_H_19_N_3_O)]
*M* *_r_* = 414.84
Triclinic, 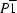

*a* = 9.9203 (2) Å
*b* = 13.5659 (2) Å
*c* = 14.6957 (2) Å
α = 112.702 (1)°
β = 91.471 (1)°
γ = 94.356 (1)°
*V* = 1815.97 (5) Å^3^
*Z* = 4
Mo *K*α radiation
μ = 1.59 mm^−1^
*T* = 100 K
0.42 × 0.33 × 0.25 mm

#### Data collection

Bruker APEXII CCD diffractometer
Absorption correction: multi-scan (*SADABS*; Sheldrick, 1996 ▶) *T*_min_ = 0.554, *T*_max_ = 0.691
15347 measured reflections
7111 independent reflections
6171 reflections with *I* > 2σ(*I*)
*R*_int_ = 0.020

#### Refinement

*R*[*F* ^2^ > 2σ(*F* ^2^)] = 0.027
*wR*(*F* ^2^) = 0.072
*S* = 1.02
7111 reflections
435 parameters
5 restraints
H-atom parameters constrained
Δρ_max_ = 0.63 e Å^−3^
Δρ_min_ = −0.27 e Å^−3^

### 

Data collection: *APEX2* (Bruker, 2007 ▶); cell refinement: *SAINT* (Bruker, 2007 ▶); data reduction: *SAINT*; program(s) used to solve structure: *SHELXS97* (Sheldrick, 2008 ▶); program(s) used to refine structure: *SHELXL97* (Sheldrick, 2008 ▶); molecular graphics: *X-SEED* (Barbour, 2001 ▶); software used to prepare material for publication: *SHELXL97* and *publCIF* (Westrip, 2010 ▶).

## Supplementary Material

Crystal structure: contains datablocks I, global. DOI: 10.1107/S1600536810053778/vm2068sup1.cif

Structure factors: contains datablocks I. DOI: 10.1107/S1600536810053778/vm2068Isup2.hkl

Additional supplementary materials:  crystallographic information; 3D view; checkCIF report

## Figures and Tables

**Table 1:** Hydrogen-bond geometry (Å, °)

*D*—H⋯*A*	*D*—H	H⋯*A*	*D*⋯*A*	*D*—H⋯*A*
C12—H12*A*⋯S1^i^	0.99	2.84	3.826 (2)	175
C16—H16⋯S2^ii^	0.95	2.74	3.670 (2)	168
C27—H27*B*⋯N9	0.99	2.55	3.421 (3)	147

Symmetry codes: (i) 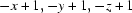; (ii) 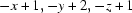.

**Fig. 1.**
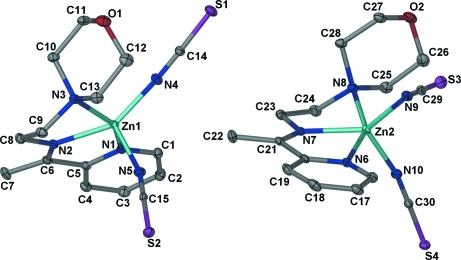
Thermal ellipsoid plot of the title compound at the 30% probability level. Hydrogen atoms have been omitted for clarity.

## supplementary crystallographic information

### Comment

2-morpholino-*N*-[1-(2-pyridyl)ethylidene]ethanamine zinc is the condensation product of the reaction of 2-acetylpyridine and 4-(2-aminoethyl)morpholine. Owing to the presence of several donor atoms and the flexibility of the morpholine ring, this Schiff base can, in principle, show ambidentate coordination behavior toward metal ions. On the other hand, thiocyanate is known to bind metal ions in different modes: *N*-donor, *S*-donor or *N:S*-bridging mode. The title compound is a mixed-ligand zinc(II) complex with the two ambidentate ligands. In the crystal, two geometrically slightly different molecules exist. The weighted r.m.s. fit for the superposition of the non-H atoms in both molecules is 1.5267 Å. The Schiff base ligand in the molecules acts as an *N,N',N"*-tridentate chelate, along with the N atoms of two isothiocyanate ligands this makes penta-coordiante zinc(II) complexes. Similar coordination environment has been reported in related mixed-ligand zinc(II) complexes (Cai, 2009; Chen *et al.*, 2005). The geometry of the two present complexes can be determined by using the index τ = (β-α)/60, where β is the largest angle and α is the second one around the metal center. For an ideal square-pyramid τ is 0, while it is 1 in a perfect trigonal-bipyramid (Addison *et al.*,1984). The τ values in the two molecules are 0.14 for the Zn1 complex and 0.33 for the Zn2 complex, indicating distorted square-pyramidal geometries. The NCS groups are almost linear [178.3 (2)° and 179.9 (3)° in the Zn1 complex; 179.1 (2)° and 179.1 (2)° in the Zn2 complex], whereas the Zn—N—CS linkages are somewhat bent [158.34 (19)° and 165.19 (18)° in the Zn1 complex; 145.54 (17)° and 169.54 (18)° in the Zn2 complex]. The morpholine rings in both molecules adopt a chair conformation. The crystal structure is stabilized by intermolecular C—H···S and an intramolecular C—H···N hydrogen bond (Table 1).

### Experimental

A mixture of 2-acetylpyridine (0.20 g, 1.65 mmol) and 4-(2-aminoethyl)morpholine (0.21 g, 1.65 mmol) in ethanol (20 ml) was refluxed for 2 hr followed by addition of a solution of zinc(II) acetate dihydrate (0.36 g, 1.65 mmol) and sodium thiocyanate (0.134 g, 1.65 mmol) in a minimum amount of water. The resulting solution was refluxed for 30 min, then left at room temperature. The crystals of the title complex were obtained in a few days.

### Refinement

The hydrogen atoms were placed at calculated positions (H—C*_Ar_* = 0.95 Å; *H*—*C_Methyl_ =* 0.98 Å; *H*—*C_Methylene_ =* 0.99 Å) and were treated as riding on their parent atoms with *U*(H) set to 1.2–1.5 *Ueq*(C). Additional rigid-bond type restraints (DELU in *SHELXL97*) were placed on the displacement parameters of S1 and C14; S2 and C15; S3 and C29; S4 and C30; N10 and C3.

### Crystal data

**Table:**

[Zn(NCS)_2_(C_13_H_19_N_3_O)]	*Z* = 4
*M**_r_* = 414.84	*F*(000) = 856
Triclinic, *P*1	*D*_x_ = 1.517 Mg m^−^^3^
Hall symbol: -P 1	Mo *K*α radiation, λ = 0.71073 Å
*a* = 9.9203 (2) Å	Cell parameters from 7261 reflections
*b* = 13.5659 (2) Å	θ = 2.5–30.5°
*c* = 14.6957 (2) Å	µ = 1.59 mm^−^^1^
α = 112.702 (1)°	*T* = 100 K
β = 91.471 (1)°	Block, yellow
γ = 94.356 (1)°	0.42 × 0.33 × 0.25 mm
*V* = 1815.97 (5) Å^3^	

### Data collection

**Table:**

Bruker APEXII CCD diffractometer	7111 independent reflections
Radiation source: fine-focus sealed tube	6171 reflections with *I* > 2σ(*I*)
graphite	*R*_int_ = 0.020
φ and ω scans	θ_max_ = 26.0°, θ_min_ = 2.1°
Absorption correction: multi-scan (*SADABS*; Sheldrick, 1996)	*h* = −12→12
*T*_min_ = 0.554, *T*_max_ = 0.691	*k* = −16→16
15347 measured reflections	*l* = −17→18

### Refinement

**Table:**

Refinement on *F*^2^	Primary atom site location: structure-invariant direct methods
Least-squares matrix: full	Secondary atom site location: difference Fourier map
*R*[*F*^2^ > 2σ(*F*^2^)] = 0.027	Hydrogen site location: inferred from neighbouring sites
*wR*(*F*^2^) = 0.072	H-atom parameters constrained
*S* = 1.02	*w* = 1/[σ^2^(*F*_o_^2^) + (0.0368*P*)^2^ + 0.8172*P*] where *P* = (*F*_o_^2^ + 2*F*_c_^2^)/3
7111 reflections	(Δ/σ)_max_ = 0.001
435 parameters	Δρ_max_ = 0.63 e Å^−^^3^
5 restraints	Δρ_min_ = −0.27 e Å^−^^3^

### Special details

**Table:**

Geometry. All e.s.d.'s (except the e.s.d. in the dihedral angle between two l.s. planes) are estimated using the full covariance matrix. The cell e.s.d.'s are taken into account individually in the estimation of e.s.d.'s in distances, angles and torsion angles; correlations between e.s.d.'s in cell parameters are only used when they are defined by crystal symmetry. An approximate (isotropic) treatment of cell e.s.d.'s is used for estimating e.s.d.'s involving l.s. planes.
Refinement. Refinement of *F*^2^ against ALL reflections. The weighted *R*-factor *wR* and goodness of fit *S* are based on *F*^2^, conventional *R*-factors *R* are based on *F*, with *F* set to zero for negative *F*^2^. The threshold expression of *F*^2^ > σ(*F*^2^) is used only for calculating *R*-factors(gt) *etc*. and is not relevant to the choice of reflections for refinement. *R*-factors based on *F*^2^ are statistically about twice as large as those based on *F*, and *R*- factors based on ALL data will be even larger.

### Fractional atomic coordinates and isotropic or equivalent isotropic displacement parameters (Å^2^)

**Table:**

	*x*	*y*	*z*	*U*_iso_*/*U*_eq_	
Zn1	0.19481 (2)	0.410155 (19)	0.190924 (17)	0.01900 (7)	
S1	0.18204 (6)	0.44706 (5)	0.52312 (4)	0.03297 (15)	
S2	0.49821 (6)	0.67240 (5)	0.15470 (4)	0.02846 (13)	
O1	0.49220 (16)	0.27533 (15)	0.32934 (13)	0.0348 (4)	
N1	−0.00833 (17)	0.43646 (14)	0.15397 (12)	0.0199 (4)	
N2	0.15687 (17)	0.30747 (14)	0.04303 (12)	0.0203 (4)	
N3	0.37745 (17)	0.31359 (15)	0.16386 (13)	0.0220 (4)	
N4	0.15900 (18)	0.42921 (16)	0.32803 (13)	0.0258 (4)	
C1	−0.0848 (2)	0.50901 (18)	0.21249 (16)	0.0250 (5)	
H1	−0.0550	0.5474	0.2799	0.030*	
C2	−0.2065 (2)	0.53031 (19)	0.17825 (18)	0.0295 (5)	
H2	−0.2585	0.5832	0.2213	0.035*	
C3	−0.2508 (2)	0.47373 (19)	0.08096 (17)	0.0292 (5)	
H3	−0.3335	0.4873	0.0559	0.035*	
C4	−0.1727 (2)	0.39631 (19)	0.01981 (17)	0.0257 (5)	
H4	−0.2022	0.3550	−0.0472	0.031*	
C5	−0.0514 (2)	0.38077 (17)	0.05858 (15)	0.0203 (4)	
C6	0.0427 (2)	0.30383 (17)	−0.00108 (15)	0.0207 (4)	
C7	−0.0008 (2)	0.2307 (2)	−0.10502 (16)	0.0303 (5)	
H7A	0.0643	0.1770	−0.1305	0.045*	
H7B	−0.0046	0.2726	−0.1462	0.045*	
H7C	−0.0906	0.1945	−0.1065	0.045*	
C8	0.2657 (2)	0.24304 (18)	−0.00450 (16)	0.0251 (5)	
H8A	0.2741	0.2412	−0.0721	0.030*	
H8B	0.2468	0.1685	−0.0090	0.030*	
C9	0.3955 (2)	0.29495 (19)	0.05839 (16)	0.0260 (5)	
H9A	0.4692	0.2479	0.0337	0.031*	
H9B	0.4218	0.3642	0.0529	0.031*	
C10	0.3596 (2)	0.20758 (18)	0.17256 (17)	0.0258 (5)	
H10A	0.4307	0.1628	0.1369	0.031*	
H10B	0.2706	0.1705	0.1409	0.031*	
C11	0.3674 (2)	0.2191 (2)	0.27917 (17)	0.0292 (5)	
H11A	0.2912	0.2582	0.3134	0.035*	
H11B	0.3584	0.1469	0.2815	0.035*	
C12	0.5035 (2)	0.3799 (2)	0.32699 (18)	0.0331 (6)	
H12A	0.5884	0.4205	0.3637	0.040*	
H12B	0.4266	0.4192	0.3599	0.040*	
C13	0.5035 (2)	0.3728 (2)	0.22158 (18)	0.0290 (5)	
H13A	0.5125	0.4461	0.2219	0.035*	
H13B	0.5823	0.3359	0.1896	0.035*	
C14	0.1676 (2)	0.43803 (17)	0.41001 (16)	0.0214 (4)	
C15	0.3793 (2)	0.59693 (16)	0.17611 (15)	0.0194 (4)	
Zn2	0.30417 (2)	0.953591 (19)	0.691039 (17)	0.01820 (7)	
S3	0.65029 (6)	1.07952 (5)	0.92910 (4)	0.03050 (14)	
S4	0.05616 (6)	1.21315 (5)	0.63775 (4)	0.02613 (13)	
O2	0.06613 (17)	0.85681 (14)	0.89498 (12)	0.0358 (4)	
N5	0.29418 (19)	0.54316 (15)	0.19158 (14)	0.0279 (4)	
N6	0.49783 (18)	0.98236 (14)	0.63008 (13)	0.0220 (4)	
N7	0.31286 (18)	0.82075 (14)	0.56062 (12)	0.0213 (4)	
N8	0.12340 (17)	0.84559 (14)	0.69974 (13)	0.0222 (4)	
N9	0.40186 (18)	1.00133 (15)	0.82261 (13)	0.0241 (4)	
N10	0.19805 (19)	1.06809 (15)	0.68484 (14)	0.0261 (4)	
C16	0.5889 (2)	1.06648 (18)	0.67001 (16)	0.0268 (5)	
H16	0.5638	1.1284	0.7224	0.032*	
C17	0.7190 (2)	1.0676 (2)	0.63832 (17)	0.0301 (5)	
H17	0.7814	1.1295	0.6675	0.036*	
C18	0.7562 (2)	0.9774 (2)	0.56381 (17)	0.0333 (6)	
H18	0.8453	0.9755	0.5415	0.040*	
C19	0.6615 (2)	0.8887 (2)	0.52143 (17)	0.0304 (5)	
H19	0.6847	0.8256	0.4695	0.036*	
C20	0.5332 (2)	0.89417 (17)	0.55625 (15)	0.0215 (4)	
C21	0.4233 (2)	0.80383 (18)	0.51743 (15)	0.0232 (5)	
C22	0.4504 (3)	0.7028 (2)	0.43377 (17)	0.0346 (6)	
H22A	0.3674	0.6537	0.4134	0.052*	
H22B	0.5214	0.6686	0.4551	0.052*	
H22C	0.4801	0.7197	0.3780	0.052*	
C23	0.1933 (2)	0.74271 (18)	0.53134 (16)	0.0274 (5)	
H23A	0.2126	0.6777	0.5428	0.033*	
H23B	0.1678	0.7213	0.4603	0.033*	
C24	0.0797 (2)	0.79550 (19)	0.59319 (16)	0.0279 (5)	
H24A	0.0490	0.8511	0.5714	0.033*	
H24B	0.0022	0.7411	0.5831	0.033*	
C25	0.0097 (2)	0.9010 (2)	0.75502 (17)	0.0299 (5)	
H25A	−0.0732	0.8505	0.7369	0.036*	
H25B	−0.0076	0.9618	0.7362	0.036*	
C26	0.0408 (3)	0.9423 (2)	0.86506 (18)	0.0344 (6)	
H26A	0.1213	0.9954	0.8837	0.041*	
H26B	−0.0366	0.9789	0.8999	0.041*	
C27	0.1808 (2)	0.8079 (2)	0.84673 (17)	0.0297 (5)	
H27A	0.2018	0.7504	0.8690	0.036*	
H27B	0.2601	0.8621	0.8656	0.036*	
C28	0.1563 (2)	0.76110 (18)	0.73578 (16)	0.0257 (5)	
H28A	0.2382	0.7286	0.7048	0.031*	
H28B	0.0805	0.7038	0.7163	0.031*	
C29	0.5052 (2)	1.03434 (17)	0.86717 (15)	0.0221 (4)	
C30	0.1385 (2)	1.12873 (17)	0.66588 (15)	0.0195 (4)	

### Atomic displacement parameters (Å^2^)

**Table:**

	*U*^11^	*U*^22^	*U*^33^	*U*^12^	*U*^13^	*U*^23^
Zn1	0.01919 (13)	0.02094 (13)	0.01724 (13)	−0.00064 (10)	−0.00103 (9)	0.00842 (10)
S1	0.0416 (3)	0.0372 (3)	0.0187 (3)	−0.0095 (3)	−0.0067 (2)	0.0122 (2)
S2	0.0309 (3)	0.0258 (3)	0.0314 (3)	0.0019 (2)	0.0092 (2)	0.0139 (2)
O1	0.0272 (9)	0.0481 (11)	0.0368 (10)	0.0044 (8)	−0.0064 (7)	0.0254 (9)
N1	0.0206 (9)	0.0204 (9)	0.0189 (9)	−0.0010 (7)	−0.0006 (7)	0.0085 (7)
N2	0.0224 (9)	0.0217 (9)	0.0178 (9)	0.0009 (7)	0.0016 (7)	0.0090 (7)
N3	0.0185 (9)	0.0266 (10)	0.0238 (9)	0.0001 (7)	−0.0003 (7)	0.0135 (8)
N4	0.0226 (9)	0.0338 (11)	0.0218 (10)	0.0031 (8)	0.0016 (8)	0.0115 (8)
C1	0.0268 (11)	0.0226 (11)	0.0227 (11)	0.0014 (9)	0.0001 (9)	0.0060 (9)
C2	0.0265 (12)	0.0270 (12)	0.0325 (13)	0.0050 (10)	0.0035 (10)	0.0085 (10)
C3	0.0220 (11)	0.0356 (13)	0.0326 (13)	0.0024 (10)	−0.0024 (10)	0.0166 (11)
C4	0.0235 (11)	0.0305 (12)	0.0233 (11)	−0.0014 (9)	−0.0034 (9)	0.0116 (10)
C5	0.0202 (10)	0.0220 (11)	0.0201 (11)	−0.0032 (8)	−0.0008 (8)	0.0107 (9)
C6	0.0233 (11)	0.0231 (11)	0.0176 (10)	−0.0007 (9)	0.0018 (8)	0.0104 (9)
C7	0.0302 (12)	0.0398 (14)	0.0171 (11)	0.0030 (11)	−0.0005 (9)	0.0070 (10)
C8	0.0259 (11)	0.0291 (12)	0.0205 (11)	0.0062 (9)	0.0045 (9)	0.0091 (9)
C9	0.0231 (11)	0.0328 (13)	0.0259 (12)	0.0058 (9)	0.0059 (9)	0.0146 (10)
C10	0.0236 (11)	0.0276 (12)	0.0301 (12)	0.0033 (9)	0.0014 (9)	0.0154 (10)
C11	0.0285 (12)	0.0334 (13)	0.0335 (13)	0.0038 (10)	0.0003 (10)	0.0216 (11)
C12	0.0208 (11)	0.0426 (15)	0.0356 (14)	−0.0012 (10)	−0.0070 (10)	0.0163 (12)
C13	0.0187 (11)	0.0347 (13)	0.0354 (13)	0.0004 (10)	−0.0016 (9)	0.0162 (11)
C14	0.0202 (10)	0.0221 (11)	0.0208 (9)	−0.0014 (8)	−0.0018 (8)	0.0080 (9)
C15	0.0252 (10)	0.0166 (10)	0.0155 (10)	0.0041 (8)	−0.0013 (8)	0.0050 (8)
Zn2	0.01948 (13)	0.01887 (13)	0.01650 (12)	0.00159 (9)	−0.00018 (9)	0.00725 (10)
S3	0.0260 (3)	0.0318 (3)	0.0302 (3)	−0.0036 (2)	−0.0090 (2)	0.0100 (3)
S4	0.0305 (3)	0.0271 (3)	0.0246 (3)	0.0090 (2)	0.0018 (2)	0.0131 (2)
O2	0.0370 (9)	0.0446 (11)	0.0289 (9)	−0.0068 (8)	0.0065 (7)	0.0194 (8)
N5	0.0326 (11)	0.0255 (10)	0.0265 (10)	−0.0047 (8)	−0.0059 (8)	0.0130 (8)
N6	0.0259 (9)	0.0223 (9)	0.0187 (9)	0.0026 (8)	0.0026 (7)	0.0089 (8)
N7	0.0276 (10)	0.0198 (9)	0.0170 (9)	0.0005 (7)	−0.0033 (7)	0.0086 (7)
N8	0.0202 (9)	0.0255 (10)	0.0210 (9)	−0.0024 (7)	−0.0029 (7)	0.0102 (8)
N9	0.0250 (10)	0.0276 (10)	0.0198 (9)	−0.0015 (8)	−0.0007 (8)	0.0101 (8)
N10	0.0296 (10)	0.0273 (10)	0.0250 (10)	0.0069 (8)	0.0042 (8)	0.0132 (8)
C16	0.0314 (12)	0.0253 (12)	0.0235 (11)	0.0001 (10)	0.0018 (9)	0.0098 (10)
C17	0.0308 (12)	0.0376 (14)	0.0230 (12)	−0.0053 (10)	0.0011 (10)	0.0146 (10)
C18	0.0272 (12)	0.0524 (16)	0.0244 (12)	0.0039 (11)	0.0069 (10)	0.0190 (12)
C19	0.0339 (13)	0.0380 (14)	0.0199 (11)	0.0090 (11)	0.0064 (10)	0.0106 (10)
C20	0.0279 (11)	0.0256 (11)	0.0136 (10)	0.0071 (9)	0.0021 (8)	0.0095 (9)
C21	0.0334 (12)	0.0245 (11)	0.0136 (10)	0.0073 (9)	−0.0017 (9)	0.0089 (9)
C22	0.0460 (15)	0.0313 (13)	0.0214 (12)	0.0067 (11)	0.0035 (11)	0.0040 (10)
C23	0.0358 (13)	0.0253 (12)	0.0185 (11)	−0.0049 (10)	−0.0071 (9)	0.0077 (9)
C24	0.0259 (11)	0.0317 (13)	0.0240 (12)	−0.0073 (10)	−0.0087 (9)	0.0112 (10)
C25	0.0222 (11)	0.0342 (13)	0.0351 (13)	−0.0021 (10)	0.0003 (10)	0.0166 (11)
C26	0.0303 (13)	0.0373 (14)	0.0337 (13)	−0.0030 (11)	0.0070 (10)	0.0123 (11)
C27	0.0306 (12)	0.0352 (13)	0.0261 (12)	−0.0081 (10)	−0.0031 (10)	0.0172 (10)
C28	0.0256 (11)	0.0265 (12)	0.0270 (12)	−0.0061 (9)	−0.0028 (9)	0.0144 (10)
C29	0.0244 (10)	0.0232 (11)	0.0186 (10)	0.0015 (8)	0.0009 (8)	0.0084 (9)
C30	0.0208 (10)	0.0203 (10)	0.0168 (10)	0.0005 (8)	0.0024 (8)	0.0067 (8)

### Geometric parameters (Å, °)

**Table:**

Zn1—N4	1.9752 (18)	Zn2—N10	1.9702 (19)
Zn1—N5	1.9858 (19)	Zn2—N9	1.9847 (18)
Zn1—N2	2.0851 (17)	Zn2—N7	2.0724 (18)
Zn1—N1	2.1647 (17)	Zn2—N6	2.2113 (18)
Zn1—N3	2.2698 (18)	Zn2—N8	2.2662 (17)
S1—C14	1.621 (2)	S3—C29	1.626 (2)
S2—C15	1.620 (2)	S4—C30	1.625 (2)
O1—C11	1.426 (3)	O2—C27	1.424 (3)
O1—C12	1.428 (3)	O2—C26	1.426 (3)
N1—C1	1.333 (3)	N6—C16	1.328 (3)
N1—C5	1.349 (3)	N6—C20	1.346 (3)
N2—C6	1.278 (3)	N7—C21	1.272 (3)
N2—C8	1.459 (3)	N7—C23	1.464 (3)
N3—C13	1.481 (3)	N8—C25	1.484 (3)
N3—C9	1.489 (3)	N8—C24	1.483 (3)
N3—C10	1.490 (3)	N8—C28	1.489 (3)
N4—C14	1.164 (3)	N9—C29	1.162 (3)
C1—C2	1.389 (3)	N10—C30	1.160 (3)
C1—H1	0.9500	C16—C17	1.384 (3)
C2—C3	1.377 (3)	C16—H16	0.9500
C2—H2	0.9500	C17—C18	1.374 (3)
C3—C4	1.393 (3)	C17—H17	0.9500
C3—H3	0.9500	C18—C19	1.392 (3)
C4—C5	1.383 (3)	C18—H18	0.9500
C4—H4	0.9500	C19—C20	1.380 (3)
C5—C6	1.488 (3)	C19—H19	0.9500
C6—C7	1.495 (3)	C20—C21	1.497 (3)
C7—H7A	0.9800	C21—C22	1.495 (3)
C7—H7B	0.9800	C22—H22A	0.9800
C7—H7C	0.9800	C22—H22B	0.9800
C8—C9	1.518 (3)	C22—H22C	0.9800
C8—H8A	0.9900	C23—C24	1.511 (3)
C8—H8B	0.9900	C23—H23A	0.9900
C9—H9A	0.9900	C23—H23B	0.9900
C9—H9B	0.9900	C24—H24A	0.9900
C10—C11	1.513 (3)	C24—H24B	0.9900
C10—H10A	0.9900	C25—C26	1.508 (3)
C10—H10B	0.9900	C25—H25A	0.9900
C11—H11A	0.9900	C25—H25B	0.9900
C11—H11B	0.9900	C26—H26A	0.9900
C12—C13	1.514 (3)	C26—H26B	0.9900
C12—H12A	0.9900	C27—C28	1.509 (3)
C12—H12B	0.9900	C27—H27A	0.9900
C13—H13A	0.9900	C27—H27B	0.9900
C13—H13B	0.9900	C28—H28A	0.9900
C15—N5	1.158 (3)	C28—H28B	0.9900
			
N4—Zn1—N5	109.20 (8)	N10—Zn2—N7	116.72 (7)
N4—Zn1—N2	144.10 (7)	N9—Zn2—N7	132.79 (7)
N5—Zn1—N2	106.50 (7)	N10—Zn2—N6	103.26 (7)
N4—Zn1—N1	94.84 (7)	N9—Zn2—N6	88.45 (7)
N5—Zn1—N1	99.47 (7)	N7—Zn2—N6	74.79 (7)
N2—Zn1—N1	75.52 (7)	N10—Zn2—N8	95.58 (7)
N4—Zn1—N3	101.50 (7)	N9—Zn2—N8	104.11 (7)
N5—Zn1—N3	95.61 (7)	N7—Zn2—N8	78.69 (7)
N2—Zn1—N3	78.47 (7)	N6—Zn2—N8	152.32 (7)
N1—Zn1—N3	152.73 (7)	C27—O2—C26	108.42 (17)
C11—O1—C12	108.95 (17)	C15—N5—Zn1	158.34 (19)
C1—N1—C5	118.96 (18)	C16—N6—C20	118.93 (19)
C1—N1—Zn1	126.23 (14)	C16—N6—Zn2	126.84 (15)
C5—N1—Zn1	114.37 (14)	C20—N6—Zn2	112.82 (14)
C6—N2—C8	123.61 (18)	C21—N7—C23	123.23 (19)
C6—N2—Zn1	119.77 (15)	C21—N7—Zn2	120.06 (15)
C8—N2—Zn1	116.62 (13)	C23—N7—Zn2	116.11 (14)
C13—N3—C9	107.76 (16)	C25—N8—C24	108.46 (17)
C13—N3—C10	108.19 (17)	C25—N8—C28	108.55 (17)
C9—N3—C10	108.54 (17)	C24—N8—C28	109.78 (17)
C13—N3—Zn1	114.89 (14)	C25—N8—Zn2	115.82 (13)
C9—N3—Zn1	99.80 (12)	C24—N8—Zn2	98.72 (13)
C10—N3—Zn1	116.86 (13)	C28—N8—Zn2	114.90 (13)
C14—N4—Zn1	165.19 (18)	C29—N9—Zn2	145.54 (17)
N1—C1—C2	122.2 (2)	C30—N10—Zn2	169.54 (18)
N1—C1—H1	118.9	N6—C16—C17	122.6 (2)
C2—C1—H1	118.9	N6—C16—H16	118.7
C3—C2—C1	119.0 (2)	C17—C16—H16	118.7
C3—C2—H2	120.5	C18—C17—C16	118.7 (2)
C1—C2—H2	120.5	C18—C17—H17	120.6
C2—C3—C4	119.1 (2)	C16—C17—H17	120.6
C2—C3—H3	120.5	C17—C18—C19	119.1 (2)
C4—C3—H3	120.5	C17—C18—H18	120.4
C5—C4—C3	118.7 (2)	C19—C18—H18	120.4
C5—C4—H4	120.7	C20—C19—C18	118.7 (2)
C3—C4—H4	120.7	C20—C19—H19	120.6
N1—C5—C4	122.0 (2)	C18—C19—H19	120.6
N1—C5—C6	114.78 (18)	N6—C20—C19	121.9 (2)
C4—C5—C6	123.18 (19)	N6—C20—C21	114.45 (18)
N2—C6—C5	115.26 (18)	C19—C20—C21	123.7 (2)
N2—C6—C7	125.6 (2)	N7—C21—C22	125.4 (2)
C5—C6—C7	119.15 (18)	N7—C21—C20	115.53 (19)
C6—C7—H7A	109.5	C22—C21—C20	119.0 (2)
C6—C7—H7B	109.5	C21—C22—H22A	109.5
H7A—C7—H7B	109.5	C21—C22—H22B	109.5
C6—C7—H7C	109.5	H22A—C22—H22B	109.5
H7A—C7—H7C	109.5	C21—C22—H22C	109.5
H7B—C7—H7C	109.5	H22A—C22—H22C	109.5
N2—C8—C9	107.21 (18)	H22B—C22—H22C	109.5
N2—C8—H8A	110.3	N7—C23—C24	107.55 (18)
C9—C8—H8A	110.3	N7—C23—H23A	110.2
N2—C8—H8B	110.3	C24—C23—H23A	110.2
C9—C8—H8B	110.3	N7—C23—H23B	110.2
H8A—C8—H8B	108.5	C24—C23—H23B	110.2
N3—C9—C8	110.92 (17)	H23A—C23—H23B	108.5
N3—C9—H9A	109.5	N8—C24—C23	111.46 (18)
C8—C9—H9A	109.5	N8—C24—H24A	109.3
N3—C9—H9B	109.5	C23—C24—H24A	109.3
C8—C9—H9B	109.5	N8—C24—H24B	109.3
H9A—C9—H9B	108.0	C23—C24—H24B	109.3
N3—C10—C11	111.94 (19)	H24A—C24—H24B	108.0
N3—C10—H10A	109.2	N8—C25—C26	111.31 (19)
C11—C10—H10A	109.2	N8—C25—H25A	109.4
N3—C10—H10B	109.2	C26—C25—H25A	109.4
C11—C10—H10B	109.2	N8—C25—H25B	109.4
H10A—C10—H10B	107.9	C26—C25—H25B	109.4
O1—C11—C10	111.59 (18)	H25A—C25—H25B	108.0
O1—C11—H11A	109.3	O2—C26—C25	111.0 (2)
C10—C11—H11A	109.3	O2—C26—H26A	109.4
O1—C11—H11B	109.3	C25—C26—H26A	109.4
C10—C11—H11B	109.3	O2—C26—H26B	109.4
H11A—C11—H11B	108.0	C25—C26—H26B	109.4
O1—C12—C13	110.8 (2)	H26A—C26—H26B	108.0
O1—C12—H12A	109.5	O2—C27—C28	111.75 (19)
C13—C12—H12A	109.5	O2—C27—H27A	109.3
O1—C12—H12B	109.5	C28—C27—H27A	109.3
C13—C12—H12B	109.5	O2—C27—H27B	109.3
H12A—C12—H12B	108.1	C28—C27—H27B	109.3
N3—C13—C12	111.49 (18)	H27A—C27—H27B	107.9
N3—C13—H13A	109.3	N8—C28—C27	110.69 (19)
C12—C13—H13A	109.3	N8—C28—H28A	109.5
N3—C13—H13B	109.3	C27—C28—H28A	109.5
C12—C13—H13B	109.3	N8—C28—H28B	109.5
H13A—C13—H13B	108.0	C27—C28—H28B	109.5
N4—C14—S1	178.3 (2)	H28A—C28—H28B	108.1
N5—C15—S2	179.9 (3)	N9—C29—S3	179.5 (2)
N10—Zn2—N9	109.96 (8)	N10—C30—S4	179.1 (2)

### Hydrogen-bond geometry (Å, °)

**Table:**

*D*—H···*A*	*D*—H	H···*A*	*D*···*A*	*D*—H···*A*
C12—H12A···S1^i^	0.99	2.84	3.826 (2)	175
C16—H16···S2^ii^	0.95	2.74	3.670 (2)	168
C27—H27B···N9	0.99	2.55	3.421 (3)	147

Symmetry codes: (i) −*x*+1, −*y*+1, −*z*+1; (ii) −*x*+1, −*y*+2, −*z*+1.
